# Planar-integrated single-crystalline perovskite photodetectors

**DOI:** 10.1038/ncomms9724

**Published:** 2015-11-09

**Authors:** Makhsud I. Saidaminov, Valerio Adinolfi, Riccardo Comin, Ahmed L. Abdelhady, Wei Peng, Ibrahim Dursun, Mingjian Yuan, Sjoerd Hoogland, Edward H. Sargent, Osman M. Bakr

**Affiliations:** 1 Division of Physical Sciences and Engineering, Solar and Photovoltaics Engineering Center, King Abdullah University of Science and Technology (KAUST), Thuwal 23955-6900, Kingdom of Saudi Arabia; 2 Department of Electrical and Computer Engineering, University of Toronto, Toronto, Ontario, Canada M5S 3G4

## Abstract

Hybrid perovskites are promising semiconductors for optoelectronic applications. However, they suffer from morphological disorder that limits their optoelectronic properties and, ultimately, device performance. Recently, perovskite single crystals have been shown to overcome this problem and exhibit impressive improvements: low trap density, low intrinsic carrier concentration, high mobility, and long diffusion length that outperform perovskite-based thin films. These characteristics make the material ideal for realizing photodetection that is simultaneously fast and sensitive; unfortunately, these macroscopic single crystals cannot be grown on a planar substrate, curtailing their potential for optoelectronic integration. Here we produce large-area planar-integrated films made up of large perovskite single crystals. These crystalline films exhibit mobility and diffusion length comparable with those of single crystals. Using this technique, we produced a high-performance light detector showing high gain (above 10^4^ electrons per photon) and high gain-bandwidth product (above 10^8^ Hz) relative to other perovskite-based optical sensors.

## Abstract (web summary)

Single crystals of organolead halide perovskites exhibit large carrier mobilities and long diffusion lengths. Here, the authors succeed in growing the single crystals on planar substrates and integrate them as the active layer of visible photodetectors with a large gain-bandwidth product.

## Graphical abstract

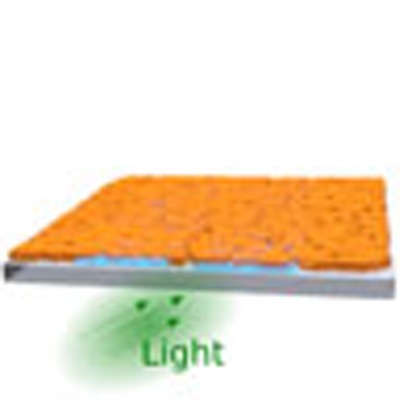

Rapid advances in perovskite materials and devices have enabled bright, widely tunable light-emitting diodes^1^,^2^, highly sensitive photodetectors^3^ and solar cells that have reached certified power conversion efficiencies above 20% (ref. 4). Hybrid perovskite-based materials are particularly attractive as they combine ready low-cost fabrication processes, such as thermal evaporation and spin coating, with remarkable electro-optical properties^5^. Perovskite solids exhibit micrometre-scale minority carrier diffusion lengths, much longer than those of most other soft materials^6^, such as organic semiconductors^7^ and colloidal quantum dot solids^8^. Through control over stoichiometry, perovskites offer tunable optical absorption and emission in the visible portion of the electromagnetic spectrum^9^.

Despite these stunning developments in material performance, the electrical properties of perovskites have yet to plateau. Whereas perovskite semiconductors are largely deposited today as thin polycrystalline films, it was recently shown that a new form of the material, consisting of large (millimetre) single crystals of hybrid perovskites, exhibit notably higher^10^,^11^ diffusion length, exceeding 5 μm, owing to vastly reduced defect densities when grain boundaries are removed. These room-temperature-grown materials reveal the extent to which a highly ordered structure is beneficial to the electrical properties of these solids.

Unfortunately, the previously reported solution-based formation of free-standing, large, single-crystal perovskite solids does not lend itself to device formation or does it enable integration with active electronic and optical devices on a planar substrate.

Here we show that single-crystal perovskites can in fact be formed on a planar substrate and over multiple cm^2^ areas, and that this integration can be exploited to build high-performance photodetectors. Specifically, we grow methylammonium lead bromide (MAPbBr_3_, where MA=CH_3_NH_3_^+^) single crystals on substrates resulting in a highly crystalline films having areas exceeding 1 cm^2^. We term the newly integrated materials planar-integrated single-crystal (ISC) perovskites. Remarkably, ISC formation does not require lattice matching with the substrate: it succeeds even on amorphous surfaces such as glass. The crystal lattice of the MAPbBr_3_ ISC is coherent over an extended length scale (>30 μm), and the electrical properties closely approach those of the best-performing free-standing single-crystal perovskites. ISC perovskites exhibit orders-of-magnitude improvements in the electrical response compared with the established polycrystalline perovskite films and traditional solution-processed materials. Excellent electrical properties are required for high-performance solution-processed optoelectronics. At present, soft-materials-based photodetectors suffer from poor electrical transport, which strongly curtails the gain-bandwidth product of the devices. We successfully integrated our high-quality ISC perovskites in a metal–semiconductor–metal photodetector. The device achieves a high signal amplification for a perovskite-based photodetector (a gain that is increased more than 25 times compared with previous reports), and also achieves a high gain-bandwidth product for a solution-processed visible light photodetector.

## Results

### Preparation and characterization of ISC perovskites

We began our study by synthesizing MAPbBr_3_ crystalline films using the antisolvent vapour-assisted crystallization technique^12^, which was demonstrated to be an effective method to grow defect-free methylammonium lead bromide single crystals^10^. Slow diffusion of antisolvent into the saturated solution resulted in the formation of a small number of seeds, and these subsequently grew by progressive incorporation of precursors from the solution, forming three-dimensional millimetre-sized crystals. Attempts to prepare ISC perovskites by implementing the antisolvent vapour-assisted crystallization technique in the presence of various substrates were unsuccessful: isotropic, that is, three-dimensional, growth of methylammonium lead bromide was always observed (Supplementary Fig. 1).

These experiments suggested that it would be necessary to develop a new method to constrain the crystallization kinetics in a two-dimensional geometry, indispensable for integrated optoelectronic device fabrication.

**Figure 1:**
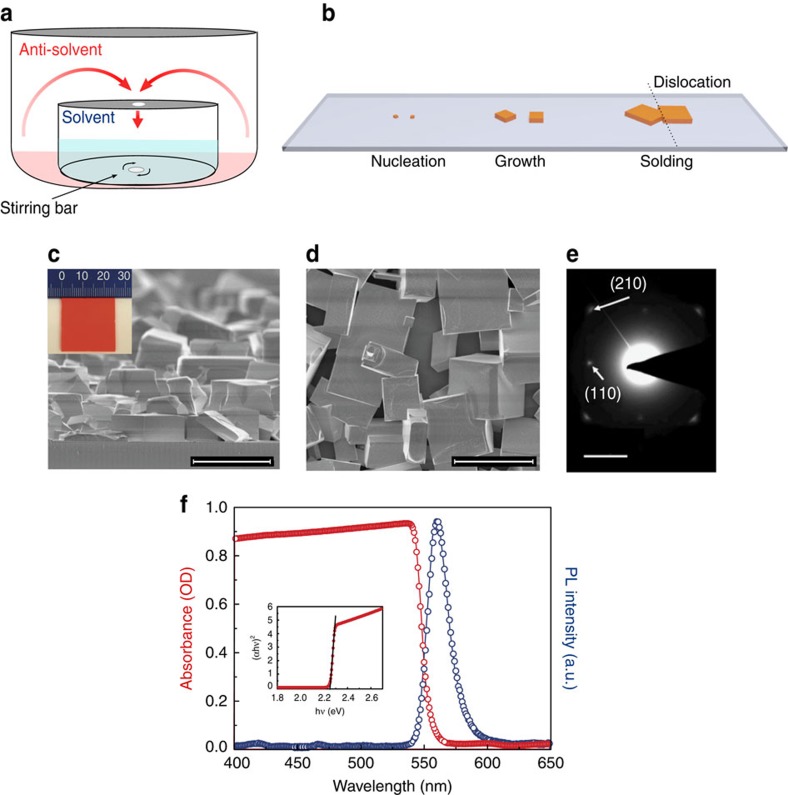
Film growth and characterization. (**a**) Schematic of the experimental procedure: a large, sealed crystallizing dish filled with antisolvent is used to host a smaller dish containing the solution. A stirring bar is placed in the inner dish. (**b**) Kinetics of ISC perovskite growth: after the initial nucleation, the crystals grow until they reach each other and merge to form a continuous solid. At the interface, the lattice continuity is lost, producing one or multiple dislocations. (**c**) Top view and (**d**) cross-sectional SEM of the ISC perovskite film. The scale bar identifies a length of 50 μm. (**e**) Electron diffraction measurements, the scale bar identifies a unit of 2 nm^−1^. (**f**) Steady-state photoluminescence and absorption. Inset: calculation of the optical bandgap using the Tauc method. The optical bandgap is measured to be *E*_g_=2.24 eV.

We took the view that a considerable increase in the number of nucleation sites would be critical to enabling the desired ISC perovskites formation. We illustrate our concept in Fig. 1a,b. At first, two nuclei are formed close to each other; the growth of the seeds continues until the two crystals reach each other and merge to form a continuous solid. We therefore aimed to generate a high and homogeneous density of nuclei, enough to cover a large area substrate with a continuous material. To create these favourable conditions, we perturbed the standard antisolvent vapour-assisted crystallization process by introducing a stirring force into the crystallization dish. After carefully optimizing process parameters (see Supplementary Figs 2–7 for details), we successfully realized a continuous, two-dimensional ISC perovskite solid.

Scanning electron microscopy (SEM) images of the optimized film showed a laterally continuous ISC perovskite solid (Fig. 1c,d): the microscopic geometry of the material indicates that crystal lattice coherence is preserved within length scales of the order of ∼30–50 μm. The crystals appear well connected to each other, whereas at the interface the relative crystal lattice phase is not preserved, likely resulting in multiple dislocations^13^.

To confirm further the high structural order of the ISC perovskites, we used electron diffraction (Fig. 1e), which revealed the presence of well-defined Bragg reflections that confirm the integrity of the crystal lattice over a wide volume^14^,^15^. X-ray diffraction analysis (Supplementary Fig. 8) applied to the ISC perovskite solid also confirmed the high purity of the MAPbBr_3_ crystal in cubic phase.

We then investigated the steady-state absorption and photoluminescence (PL) of the ISC perovskites (Fig. 1f). Analysis of the absorption profile showed that the band edge is located at 554 nm (2.24 eV). The steep rise at the band edge suggests a low concentration of in-gap defects. The PL peak appears very close to the band edge (560 nm), this low Stokes shift indicating a small vibronic relaxation^16^. These results differ materially from polycrystalline films, whose PL emission is usually located at *λ*<543 nm (refs 17, 18).

The lineshape of the PL, acquired at ambient conditions and under a humidity level of ∼57%, was monitored over 30 days showing no appreciable change (Supplementary Fig. 9); this is a strong indication of the high level of stability of the material over time.

**Figure 2:**
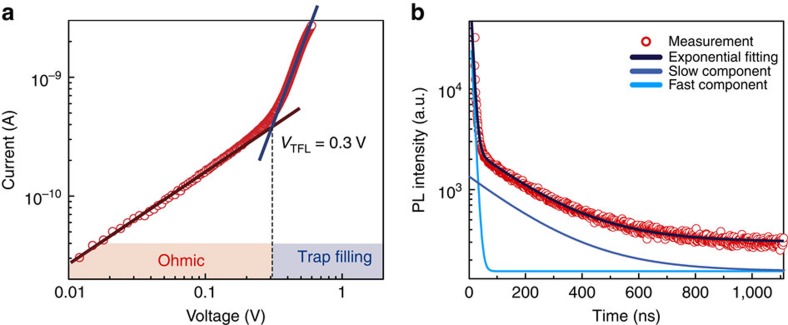
Current-voltage (IV) trace and lifetime measurements. (**a**) IV characteristic of the MAPbBr_3_ ISC perovskites showing an ohmic region followed by trap filling region starting at *V*_TFL_=0.3 V. From these data, we extract a conductivity *σ*=2 × 10^−8^ (Ω cm)^−1^ and a density of trap states *n*_trap_=2 × 10^11^ cm^−3^. (**b**) PL time decay trace at *λ*=560 nm, with bi-exponential fits showing a fast (7±1 ns) and a slow transient (189±10 ns).

PL decay traces, presented in Fig. 2b, were fit using a bi-exponential profile, from which we obtained a fast and a slow time constant of *τ*≈7 and *τ*≈189 ns, respectively. We associate these, as in prior reports, with surface and bulk recombination, respectively^10^.

### Transport properties of ISC perovskites

To evaluate the electrical performance of the MAPbBr_3_ ISC perovskites, and therefore their potential for light sensing, we performed in-depth optoelectronic characterization. First, the current–voltage (IV) characteristic of the samples was measured and analysed using the space charge limited current^19^,^20^ method. Figure 2a shows an ohmic region followed by a steep increase of the IV curve, indicating the presence of a trap-filling region starting at *V*_TFL_=0.3 V. From these data, we extracted the density of deep trap states: *n*_trap_∼2 × 10^11^ cm^−3^ estimated according to the formula *n*_trap_=2*ɛV*_TFL_/*qL*^2^ (where *ɛ* is the dielectric constant of the material, *q* the elemental charge and *L* the length of the solid under test). Analysis of the ohmic region also reveals a conductivity *σ*∼2 × 10^−8^ (Ω cm)^−1^, a value in excellent agreement with previous reports on MAPbBr_3_ free-standing single crystals^10^.

To deepen further understanding of the transport properties of the ISC perovskites, we determined the carrier mobility independently using the Hall method. We obtained a high value of *μ*=60 cm^2^ V^−1^ s^−1^ (Supplementary Fig. 10). This measurement also revealed p-type conduction with an exceedingly low free carrier concentration of *p*∼10^9^ cm^−3^; these results are again in close agreement with the estimates for free-standing single crystals^10^. A lateral spacing of ∼1.5 mm between parallel electrical contacts was used, such that an average of ∼100 grain boundaries is included along the transport path investigated. With the aid of the Einstein relation (*D*=*μ K*_B_*T*/*q, K*_B_ is the Boltzmann's constant and *T* is the sample's temperature), we estimate the diffusion length according to: 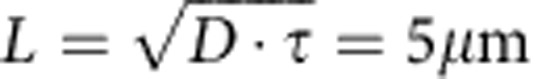.

To evaluate the quality of the ISC perovskites, we compared their measured electrical parameters with those of free-standing single crystals and polycrystalline films (Table 1). First, we noted that the trap density of the ISC perovskites is only ∼10 times higher than that of a free-standing millimetre-dimension single crystal. The higher surface/volume ratio in the ISC perovskites and the presence of crystal-to-crystal interfaces accounts for this trap density. As expected, the larger density of traps has an impact on the lifetime, which is slightly lower than in free-standing single crystals. The Hall mobility is unchanged compared with the free-standing single crystals, suggesting that the crystal-to-crystal interfaces play a significant role only in recombination, and not in transport.

The analogous analysis on MAPbBr_3_ polycrystalline films revealed a dramatic worsening of all the transport-related parameters (details about the measurements on polycrystalline films can be found in the Supplementary Figs 11 and 12). In sum, the MAPbBr_3_ ISC perovskites exhibit electrical performance comparable to free-standing single crystals, and therefore dramatically outperform traditional disordered thin film perovskites in optoelectronic figures of merit.

### Photodetectors based on ISC perovskites

The properties of the ISC perovskites appear ideal for solution processed photodetection: the high mobility has a fundamental role in producing high gain and fast response time^21^. The low free carrier concentration enables a low dark current, critical for the noise performance of detector^22^. The steep absorption edge defines precisely the window of operating light frequencies.

**Figure 3:**
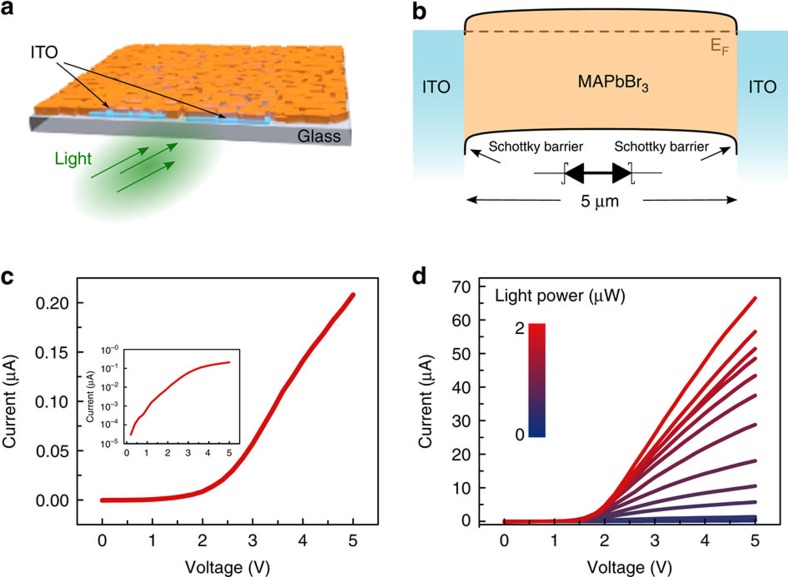
Photodetector based on ISC film. (**a**) Three-dimensional illustration of the photodetector: the MAPbBr_3_ ISC perovskite layer (orange) is deposited on top of the ITO contacts (light blue). The detector lay on a glass substrate (transparent grey). (**b**) Energy diagram of the photodetector. (**c**) Current–voltage characteristic measured in dark condition and (**d**) under different light power illumination, the colour bar is logarithmic.

We therefore conceived an ISC perovskite photodetector to realize high sensitivity and high gain-bandwidth product. The device architecture employed is depicted in Fig. 3a. Indium tin oxide (ITO) interdigitated contacts were patterned on top of a glass substrate. A MAPbBr_3_ ISC perovskite layer was then grown on top of the ITO-coated glass (Supplementary Fig. 13). The transparent glass substrate allowed for illumination from the bottom (contact) side.

As depicted in the band diagram (Fig. 3b), the ITO—MAPbBr_3_ interface is expected to produce a rectifying junction, as previously reported^23^,^24^. Two Schottky barriers contacting the active layer realize a metal—semiconductor—metal photodetector^25^. This type of detector, typically employing the same planar, interdigitated architecture used in this work, are extensively used in optoelectronics, as they combine facile fabrication with ease of integration and high performance^26^.

As expected, the measured dark IV characteristic of the device (Fig. 3c) clearly exhibits a rectifying behaviour; the inset of Fig. 3c indicates that the IV response is described by exponential behaviour up to an applied voltage of 3 V, beyond which point series resistance effects dominate^27^. The IV curve retains a rectifying shape also after applying optical excitation of variable intensity modulated over different orders of magnitude (Fig. 3d). This measurement also reveals that a considerable amount of photocurrent can be photogenerated.

**Figure 4:**
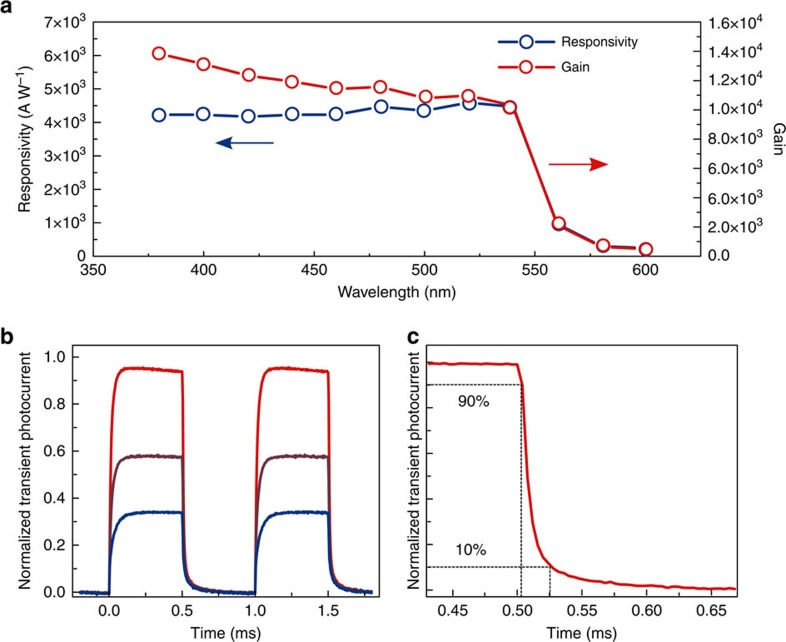
Performance of the photodetector based on ISC film. (**a**) Responsivity and gain (external quantum efficiency) of the photodetector. The device exhibited high and flat response for all the absorbed light frequency. (**b**) Transient photocurrent of the photodetector as a function of incident light power (1.1, 2.2 and 3.8 μW for the blue, brown and red trace, respectively). (**c**) The fall time of the photodetector is measured to be ∼25 μs.

We therefore proceeded to a complete characterization of the photodetector performance. In Fig. 4a the spectral responsivity and the spectral gain (defined as the external quantum efficiency) are shown. A large responsivity (defined as the ratio of the light current over the incident light power *R*=*I*_light_/*P*_in_) exceeding 4,000 A W^−1^ is measured, corresponding to a gain above 10^4^ electrons/photons. We also note that, very attractively, the responsivity remains constant over all the absorbed light frequencies, in contrast with previously reported perovskite detectors that performed well only at ultraviolet wavelengths^28^. The values of gain reported here represent, to the best of our knowledge, the highest result for a perovskite light detector to date (more than one order of magnitude higher than previous reports, Supplementary Fig. 14). We attributed such a high gain to photoconduction, a mechanism that has previously reported for perovskites/ITO junction devices^24^. Gain in detectors has been attributed in the past to shallow traps populating the metal–semiconductor interface^29^. The high responsivity is a direct consequence of the high mobility of the ISC perovskites; photoconductive gain^30^ is defined by the relation 
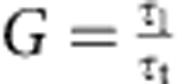
, with *τ*_1_ the charge lifetime and *τ*_t_ the charge carrier transit time, with the latter inversely proportional to the mobility of the semiconductor according to the relation 
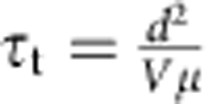
 (where *d* is sample thickness, *V* is applied voltage). To shed further light on the nature of the gain mechanism, we measured the responsivity as a function of the incident light intensity (Supplementary Fig. 15). We observed an increase of the responsivity with decreasing incident light power, eventually reaching saturation at low light intensity, a behaviour that is typical of photoconductive detectors^31^.

Last, we analysed the response time, as shown in Fig. 4b,c. A fall time (defined as the time necessary to reduce the photoresponse from 90 to 10%) as low as 25 μs was measured, confirming that ISC perovskites allow for the simultaneous realization of a sensitive and fast device. Comparable rise time is shown in Supplementary Fig. 16. Combining gain and response time we obtain a gain-bandwidth product of ∼10^8^ Hz: this figure, which compares well with industry-leading III–V phototransistors^32^,^33^, exceeds previously reported solution processed visible detectors by a factor exceeding 10 (Supplementary Fig. 14). The photodetector detectivity (D*) exceeds 10^13^ Jones, a value comparable with the most sensitive perovskites detectors presented in the literature (Supplementary Fig. 17)^3^,^34^. The noise current of the device was also measured and found to be close to the theoretical limit set by the shot noise (Supplementary Fig. 18). These results were achieved thanks to the low free carrier concentration of the ISC perovskites, which offer simultaneously a very high mobility a low dark current.

## Discussion

The approach described herein builds on the recent advent of the antisolvent vapour-assisted crystallization technique to produce large-area, planar-integrated perovskite films having exceptional electronic properties. The performance approaches that of high-purity free-standing single crystals. The ISC perovskites were used to realize a high-performance light detector showing gain, and gain-bandwidth product, outperforming previously reported perovskite-based photodetectors.

## Methods

### Chemicals and reagents

Lead bromide (PbBr_2_ 99.999%) and dimethylformamide (anhydrous, 99.8%) were purchased from Sigma-Aldrich. Methylammonium bromide (MABr) was purchased from Dyesol Limited (Australia). All salts and solvents were used as received without any further purification.

### ISC film preparation

Ten millilitres of a 0.1 M solution of PbBr_2_/MABr (1/1) in dimethylformamide were filtered using a polytetrafluoroethylene membrane filter (with a pore size of 0.2 μm), and poured into an 80-mm crystallizing dish containing the substrates. This crystallizing dish, covered with an aluminum foil with a 5-mm diameter opening allowing the antisolvent diffusion, was placed into a 120-mm crystallization dish hosting 40 ml of dichloromethane. The system was kept on the stirring plate at 400 r.p.m., at room temperature. After ∼48 h, the substrates were removed from the solution and were annealed for 5 min at 135 °C on a hot-plate.

### Characterization

The crystal structure of ISC was characterized using X-ray diffraction (CuK_a_ excitation). Steady-state PL measurements were carried out on a FLS920 spectrofluorometer by Edinburgh Instruments. The absorption spectrum of the ISC was characterized using a Varian Cary 5000 spectrometer. Quanta 600 FEG was used to acquire SEM images. The IV characteristic (used for the space charge limited current analysis) was measured using a Keithley 6430 sub-femtoamp sourcemeter. During the measurement, the sample was kept in a dark environment, under vacuum. PL decay measurements were performed using a Horiba Fluorolog Time Correlated Single Photon Counting system.

### Metal-semiconductor-metal photodetector fabrication and characterization

A glass substrate covered with a thin ITO film was cleaned using successively acetone, isopropanol and de-ionized water. Interdigitated contacts were obtained by patterning the ITO film using photolithography and subsequent wet etching in hydrochloric acid (HCl). We defined a channel length of 5 μm and a channel width of 1 mm. The MAPbBr_3_ crystalline film was deposited on top of the substrate and the contacts using the methods previously described.

The IV characteristic of the device was measured using a Keithley 2400 sourcemeter. The device was kept in a dark environment. For the light characteristic, a green monochromatic (*λ*=520 nm) source was used. The spectral responsivity of the detector was measured by using a technique previously described in literature^30^,^31^,^35^: the monochromatic light excitation was produced by using a 450-W Horiba Jobin-Yvon xenon arc lamp filtered by a monochromator; appropriate optical filters (Newport) were used and the incident light power was taken constant using a neutral density filter. The intensity profile of the incident light was integrated over the device active area to determine the incident power. The photocurrent was measured using a Standford research SRS 830 lock-in amplifier. The device was biased (*V*_DS_=5 V) using a Keithley 2400 sourcemeter. The time response of the photodetector was measured using a Tektronix TDS 5104 digital oscilloscope; the pulsed light excitation was provided using a monochromatic source (LED, *λ*=520 nm) driven by a function generator. The device was biased (*V*_DS_=20 V) using a Keithley 2400 sourcemeter.

## Additional information

**How to cite this article:** Saidaminov, M. I. *et al*. Planar-integrated single-crystalline perovskite photodetectors. *Nat. Commun.* 6:8724 doi: 10.1038/ncomms9724 (2015).

## Supplementary Material

Supplementary Information Supplementary Figures 1-18 and Supplementary References.

## Figures and Tables

**Table 1:** Comparison of transport properties.

	**Free-standing crystal**	**ISC perovskite**	**Polycrystalline film**
Mobility (cm^2^ V^−1^ s^−1^)	60	60	0.26
PL lifetime (ns)	357	189	168
Diffusion length (μm)	7.5	5	0.33
Trap density (cm^−3^)	5.8 × 10^9^	2 × 10^11^	1 × 10^17^

ISC, planar-integrated single-crystal; PL, photoluminescence.

Transport figures of merit for MAPbBr_3_ free-standing single crystals, ISC perovskites and polycrystalline thin film.
