# Supplementary material for: Planar-integrated single-crystalline perovskite photodetectors
Source: Nat Commun. 2015 Nov 9;6:8724. doi: 10.1038/ncomms9724 (PMC4667636; doi:10.1038/ncomms9724)
Supplement: Supplementary Information — Supplementary Figures 1-18 and Supplementary References. [file ncomms9724-s1.pdf]

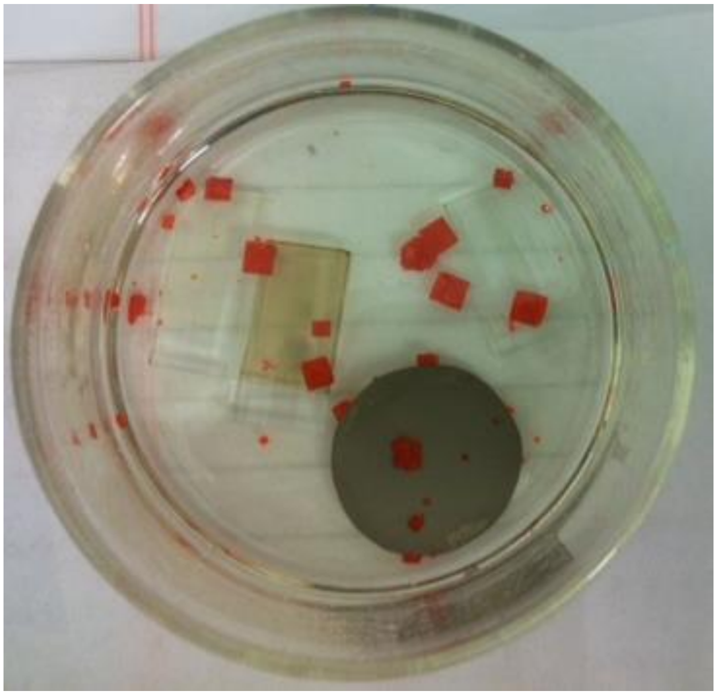

Supplementary Figure 1. Unperturbed Antisolvent Vapour-assisted Crystallization (AVC). AVC using different substrates such as FTO, $cTiO_2$/FTO, mesoporous-Al_2O_3, and mesoporous-TiO_2/FTO. In all the cases we observe the formation of free-standing millimeter sized crystals.

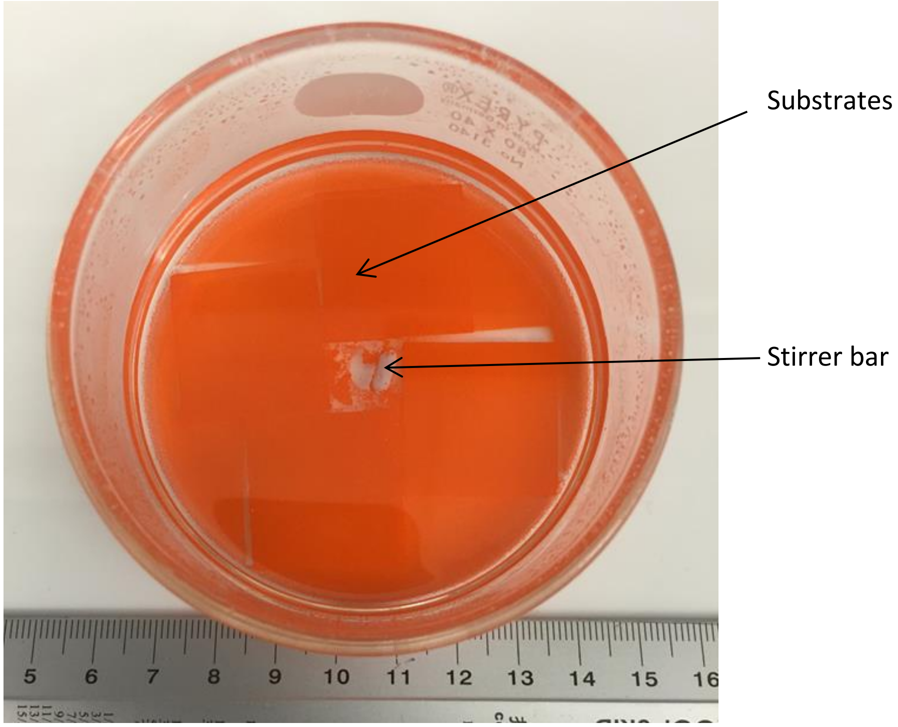

Supplementary Figure 2. Perturbation of the AVC with the stirring force. AVC perturbed using a stirring bar during the crystallization. The bottom of an 80 mm crystallization dish (~50 cm^2) is fully covered with a crystalline film. We reasoned that this would result in a more homogeneous diffusion of the antisolvent within the solution; in the subsequent formation of many nuclei; and in the ensuing growth and interconnection of the crystals. The stirring rate was optimized such that the crystals formed retain their starting positions on the substrate and grow into a continuous ISC perovskite.

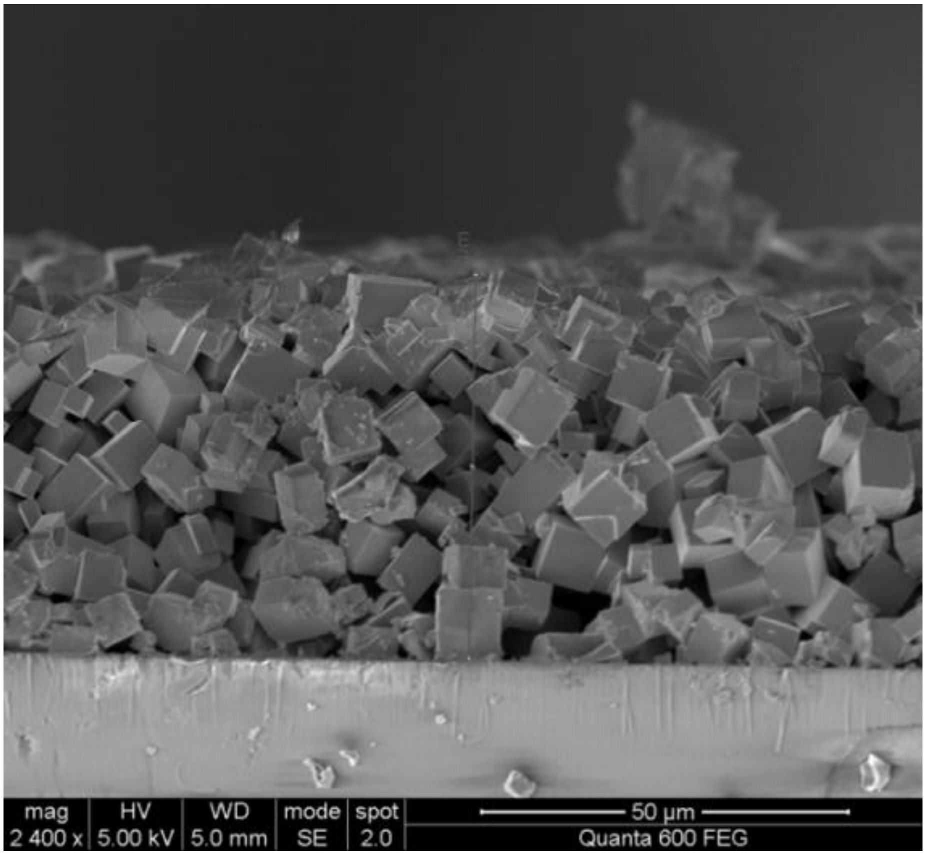

Supplementary Figure 3. The role of diameter of inner dish. Cross-sectional SEM of a film crystallized using 10 ml of 0.2 M solution. The inner dish was uncovered. Multiple layers of small crystals (5-10 μm) were formed; the crystals appear not well connected to each other. The opening diameter of the inner dish, which affects the diffusion rate of antisolvent, was found to play a crucial role: uncovered dishes result in formation of small, 5-10 μm crystals with well-defined boundaries.

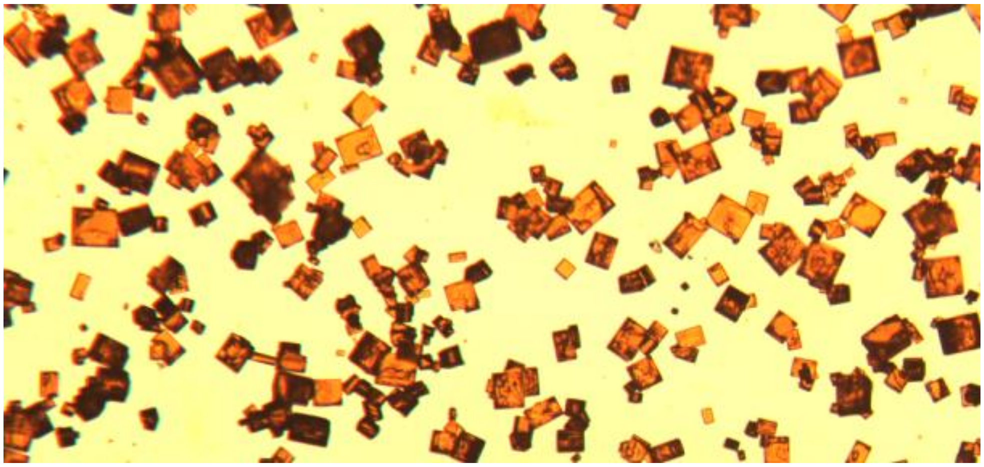

Supplementary Figure 4. The role of solution concentration. Crystals prepared from 5 ml of 0.05 M solution of MABr/$PbBr_2$ (1/1) in DMF. The crystals appear well separated each other, not forming a continuous ICS.

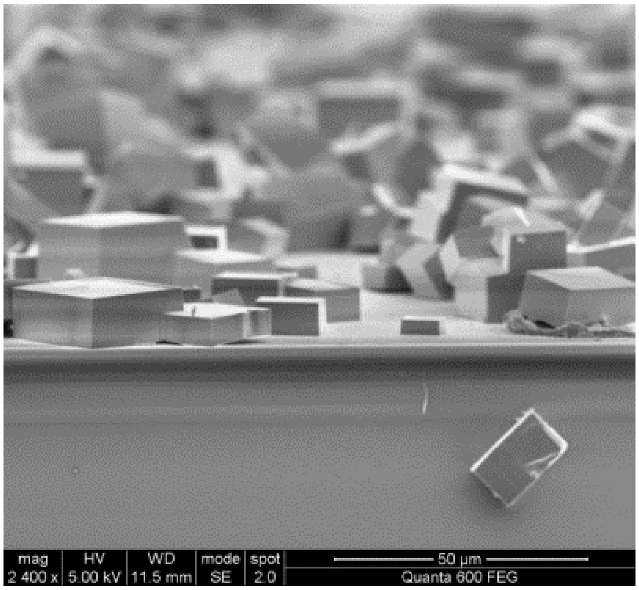

Supplementary Figure 5. The role of solution volume. Cross-sectional SEM of separated crystals prepared from 5 ml 0.1 M solution of $MABr/PbBr_2$ (1/1) in DMF.

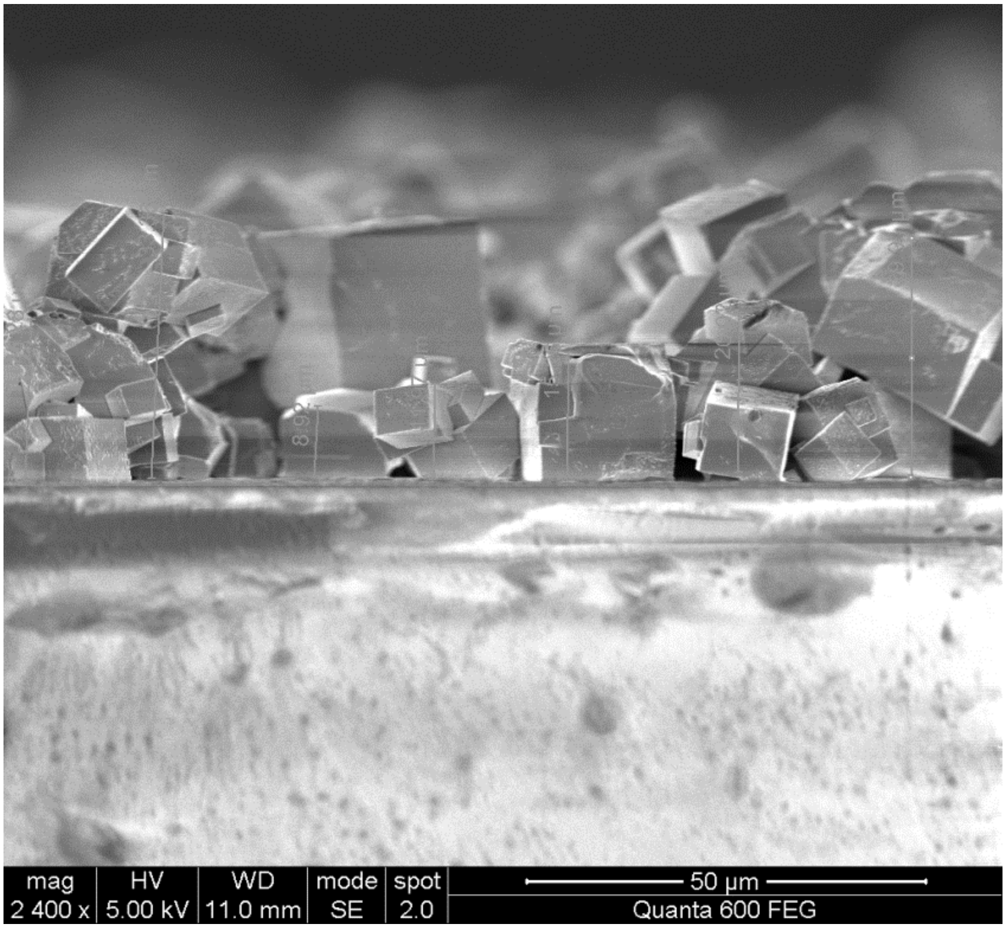

Supplementary Figure 6. The effect of solution volume. Cross-sectional SEM of the film prepared from 10 ml 0.1 M solution of $MABr/PbBr_2$ (1/1) in DMF.

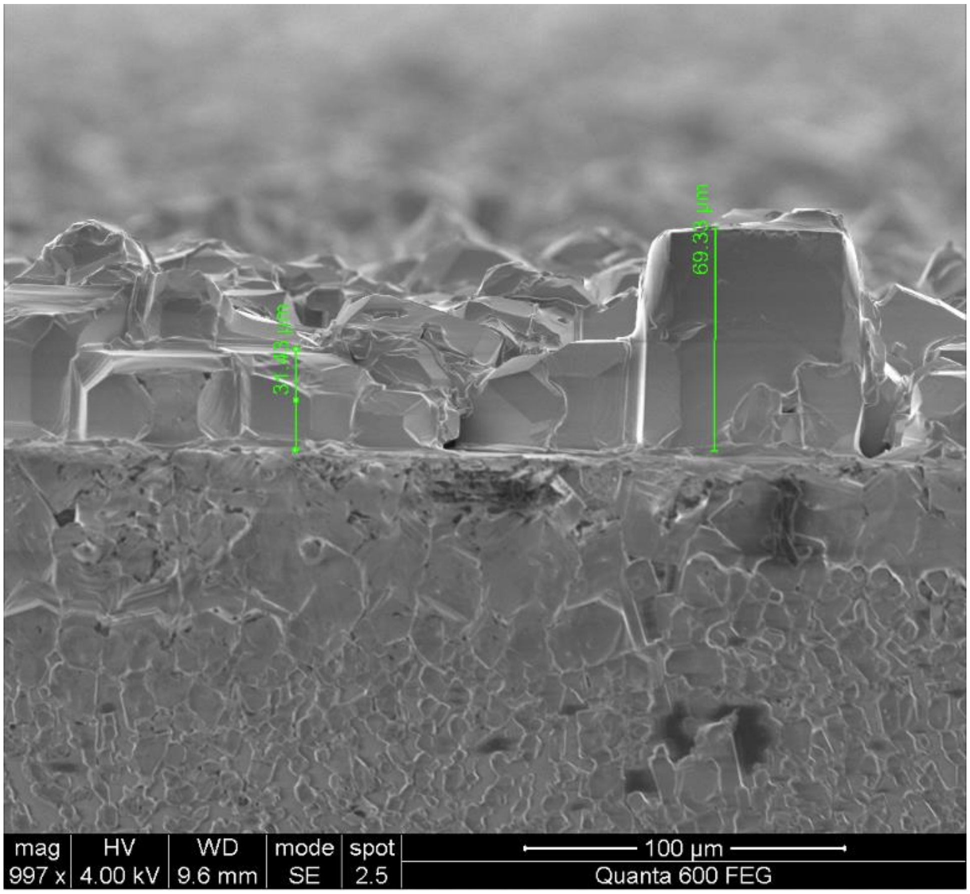

Supplementary Figure 7. The effect of solution concentration. Cross-sectional SEM of a film prepared from 10 ml 0.2 M solution of $MABr/PbBr_2$ (1/1) in DMF.

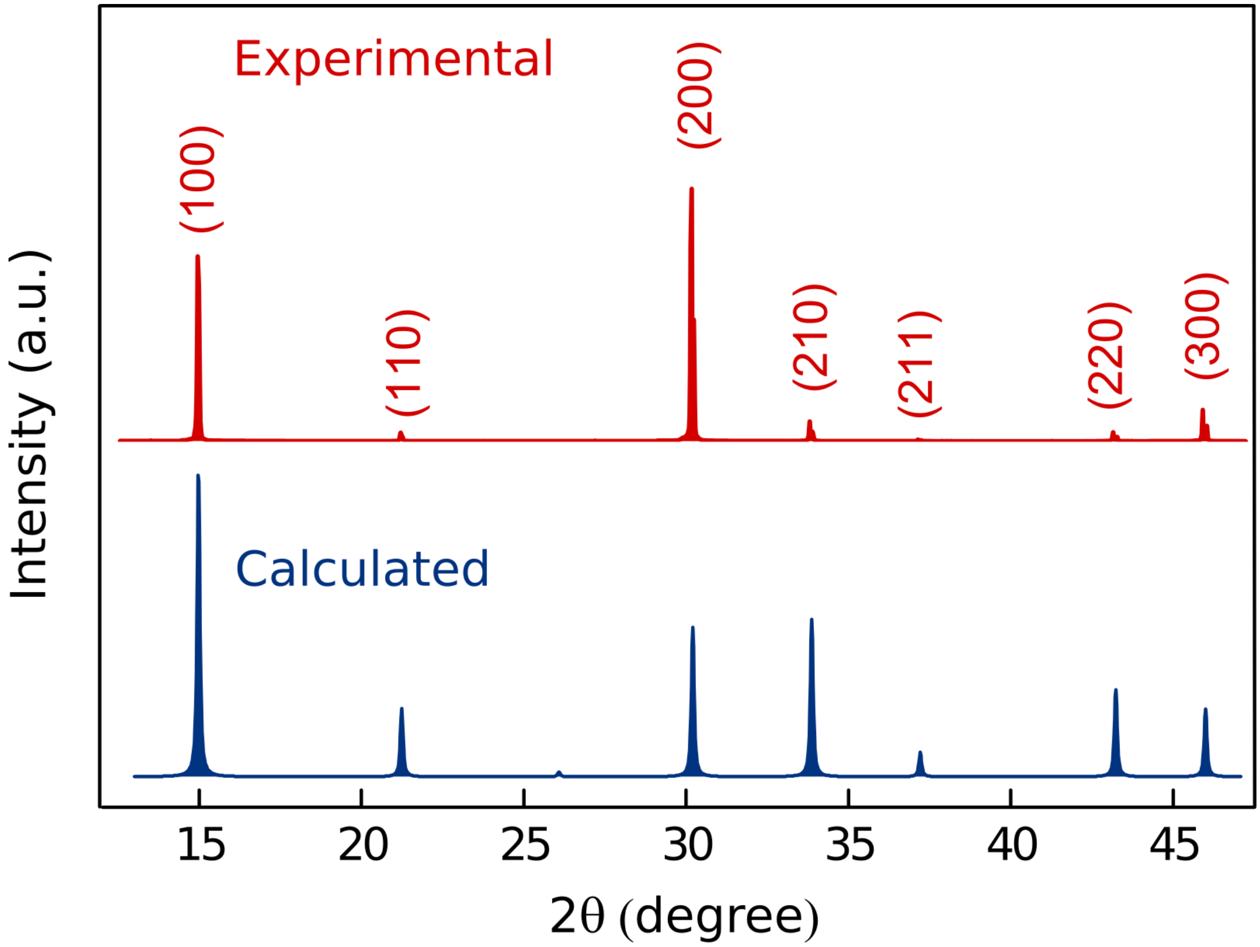

Supplementary Figure 8. X-Ray Diffraction (XRD). XRD of the $MAPbBr_3$ ISC perovskite.

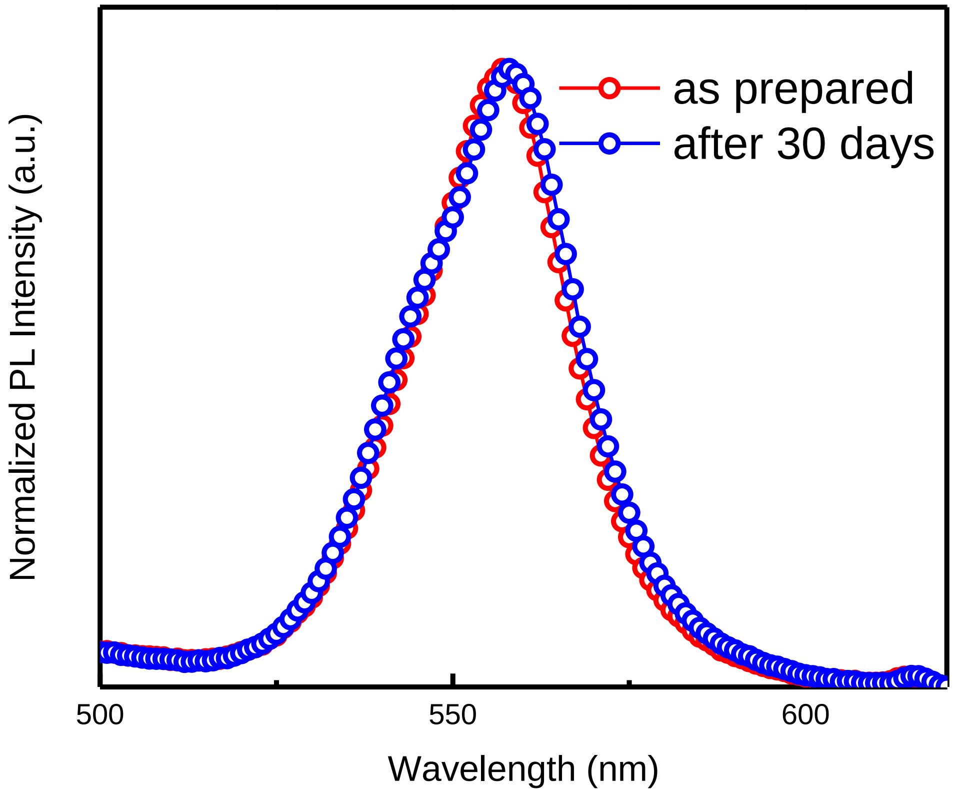

Supplementary Figure 9. **Photoluminescence stability.** Steady-state photoluminescence of the ISC perovskite: fresh (red curve) and after 30 days (blue curve).

a)

Nanometrics HL5500 Hall System
Measured on 12/09/14 at 4:11 PM

SPECIMEN	MEASURING CONDITIONS
Wafer ID:	I-meas: 0.00475 nA DC
Batch ID:	Temperature: 300.6
Material: MAPbBr3	Field: 0.504 Tesla
Description:	
Thickness:20.000 µm	Targ.Vr: 40 mV

RESULTS SUMMARY

Rs: 3.809e+10 ohm/sq	RHs: +2.28e+08 m²/C	Ns: +2.733e+06 /cm²
R : 7.618e+07 ohm-cm	Mob: 60 cm²/V-s	N : +1.367e+09 /cm³

RESISTIVITY

Meas	+++	Vm ---	Sym	Factor	R-sheet
43	+1.022e-01	+2.155e-02	1.06	1.00	3.966e+10
41	-3.074e-02	-1.164e-01	1.09	1.00	3.909e+10
21	-7.557e-02	-1.539e-01	1.05	1.00	3.654e+10
23	+3.036e-02	-4.453e-02	1.08	1.00	3.709e+10

HALL MEASUREMENTS	+++ 24	---	+++ 13	---
Misalignment	-5.54e-02	-3.54e-02	+1.02e-01	+8.40e-02
Offset applied	+6.67e-03		-6.67e-03	
V-hall North	-7.59e-02	-5.27e-02	+1.05e-01	+9.29e-02
V-hall South	-9.07e-02	-6.47e-02	+9.92e-02	+8.82e-02
V-hall (mean)	+7.09e-04		+3.84e-04	
V-hall (over all cycles)	+5.47e-04			

b)

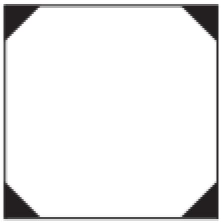

Supplementary Figure 10. Hall Effect Measurements. a) Log sheet of the Hall Effect measurement, measured with a Nanometrics HL5500 Hall system. The resistivity, measured using the 4-probes technique, confirms the findings previously reported in literature. The solid appears to be p-type, as shown from the Hall Effect coefficient **RHs**. The value of the holes mobility is particularly high (60 cm^2/Vs) while the free carrier density is found to be very small; these results confirm that the quality of the ISC perovskite closely resemble that one of free standing millimeter size $MAPbBr_3$ single crystals. The measurement conditions: magnetic field intensity (0.504 T), temperature (300.6 K), target voltage (40 mV). These conditions are noted on the log sheet (Supplementary Figure 10a). Given the high resistivity of the sample the

Nanometrics HL5500 was used in a configuration that includes a current amplifier. The samples were contacted using a MoOx/Au/Ag stack that is well known to provide a good ohmic junction with MAPbBr3 single crystals. b) The contact geometry used for the measurements (the black corners are the contacts).

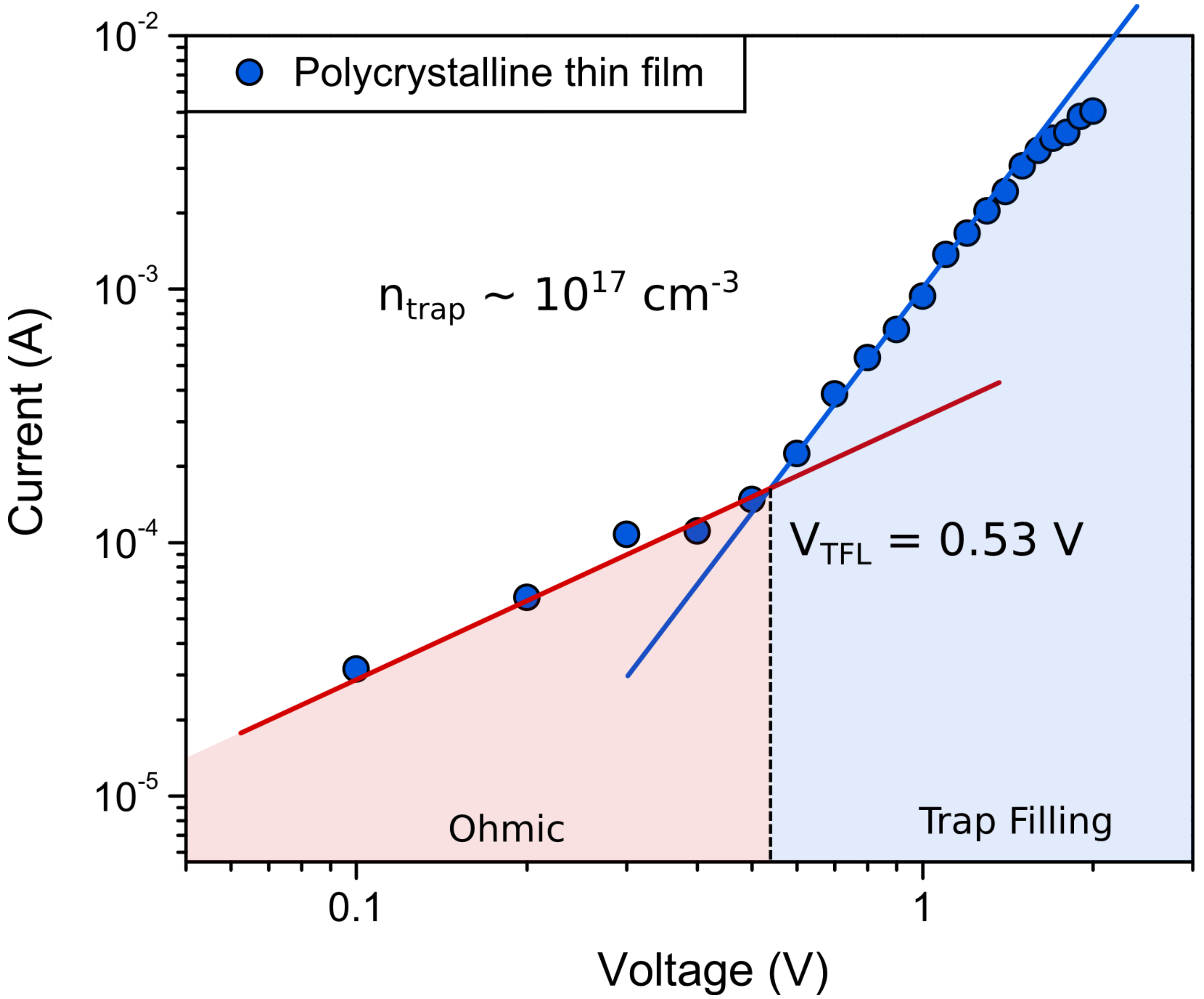

Supplementary Figure 11. Polycrystalline Film's Trap Density. IV curve and Space Charge limited Current analysis for a polycrystalline thin film (thickness ~ 100 nm). At an applied voltage of V_{TFL} = 0.53 V, a clear transition from the ohmic to trap filling regime is observed. The total concentration of traps measured for this sample was $n_{trap} \sim 10^{17}$ cm^{-3}

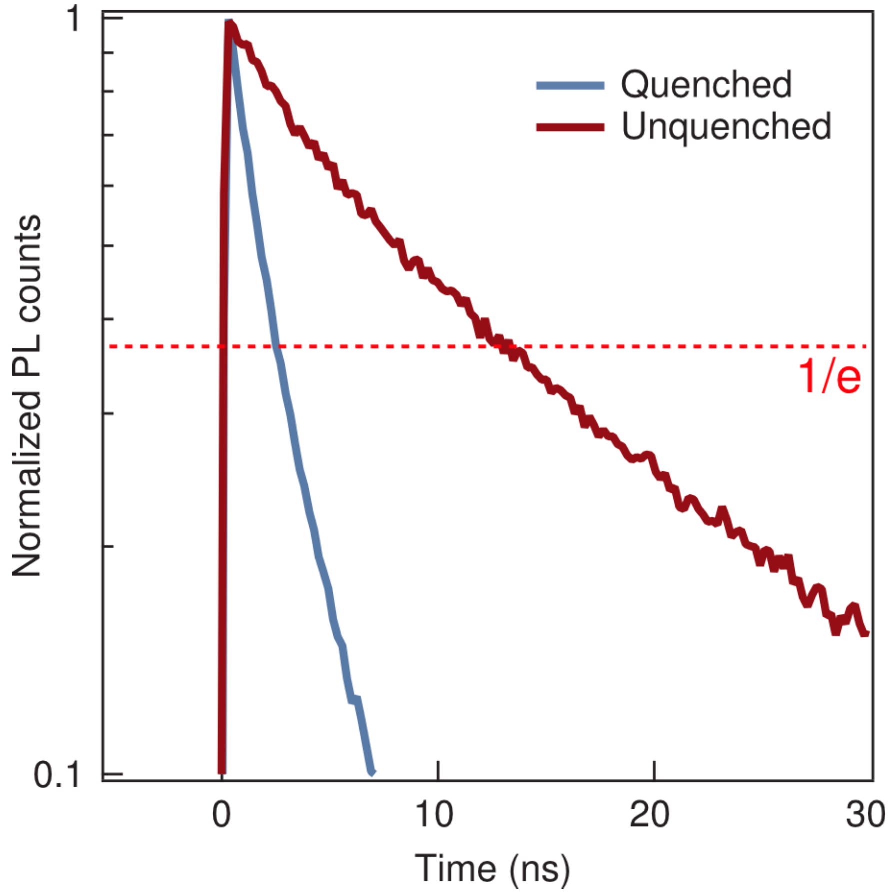

Supplementary Figure 12. PL decay and diffusion length of polycrystalline film. Photoluminescence decay for polycrystalline films: the transient signal is measured for the bare film (red curve) and in presence of a quenching layer (blue curve). The lifetime data are analyzed to extract the diffusion length of the charge carriers within the solid, using a method previously outlined in literature[1].

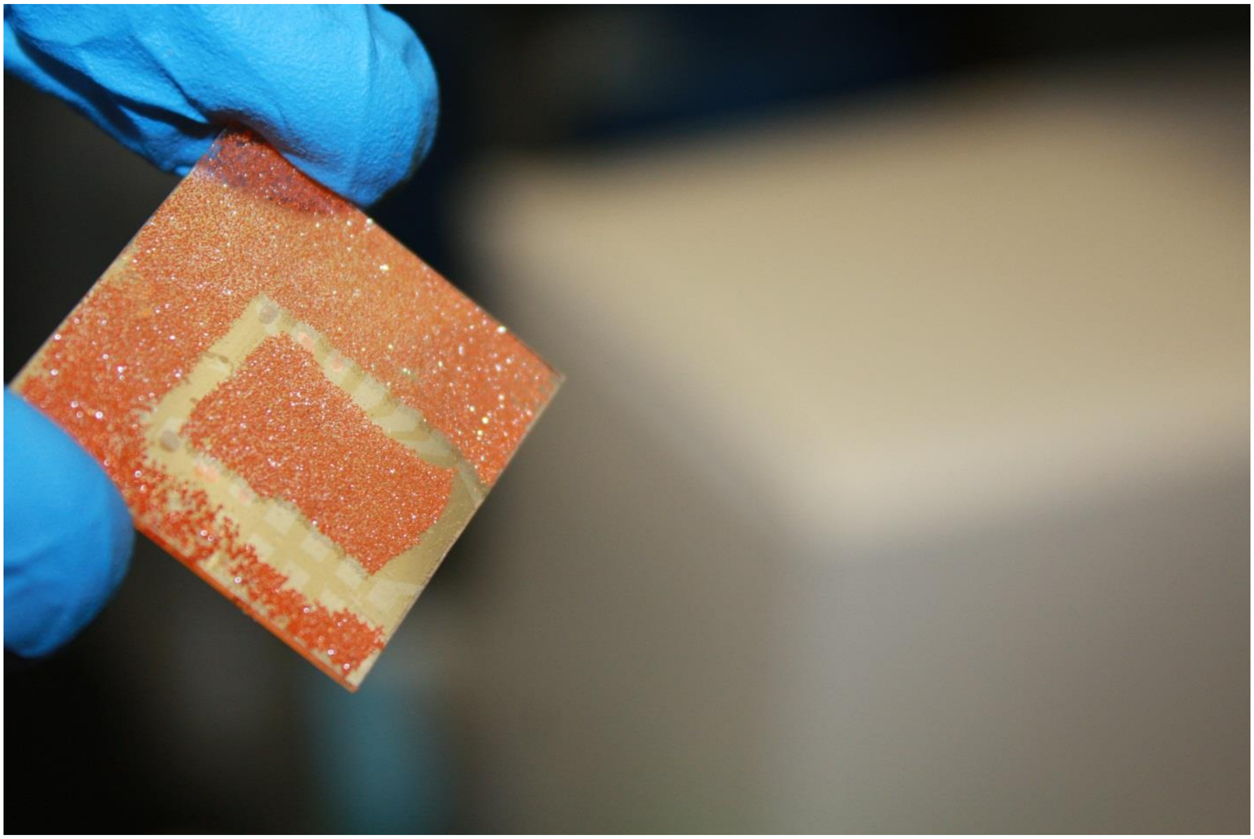

Supplementary Figure 13. Photographic picture of the device. Photographical picture of the device. Part of the film was removed in order to reveal the ITO patterning laying below the $MAPbBr_3$ crystals. The ITO pads, used to access the electrical interdigitated micro-contacts, are clearly visible.

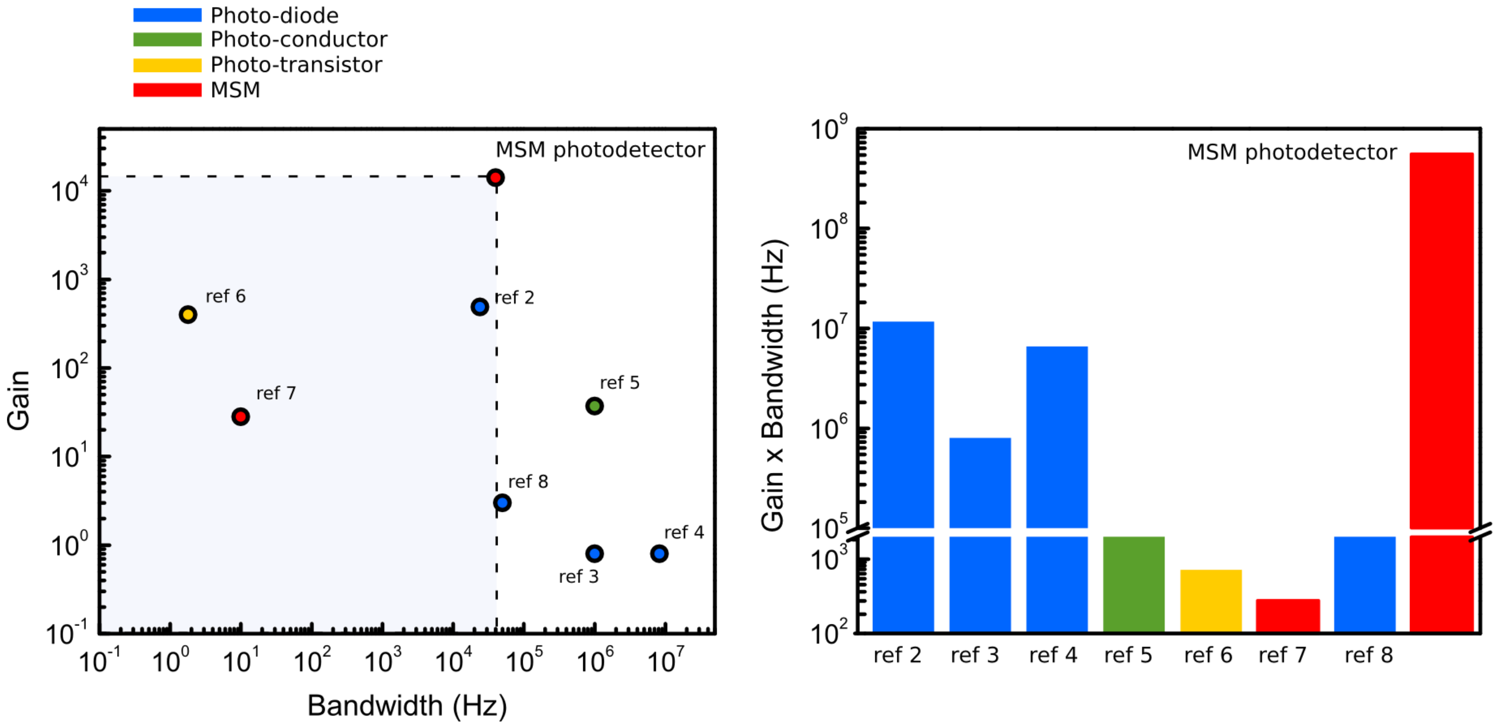

Supplementary Figure 14. Perovskite light detector review. Comparison between the metal – semiconductor – metal (MSM) photodetector presented in this work and other perovskite detectors previously reported in literature[2–8]. A color code identifies the photodetector architecture. On the left a gain-bandwidth plot is shown: the MSM photodetector clearly shows a superior gain compared to the other devices (> 10x). In the right plot the gain-bandwidth product is shown: also for this figure of merit the MSM photodetector exceeds the other devices of a factor higher then 10.

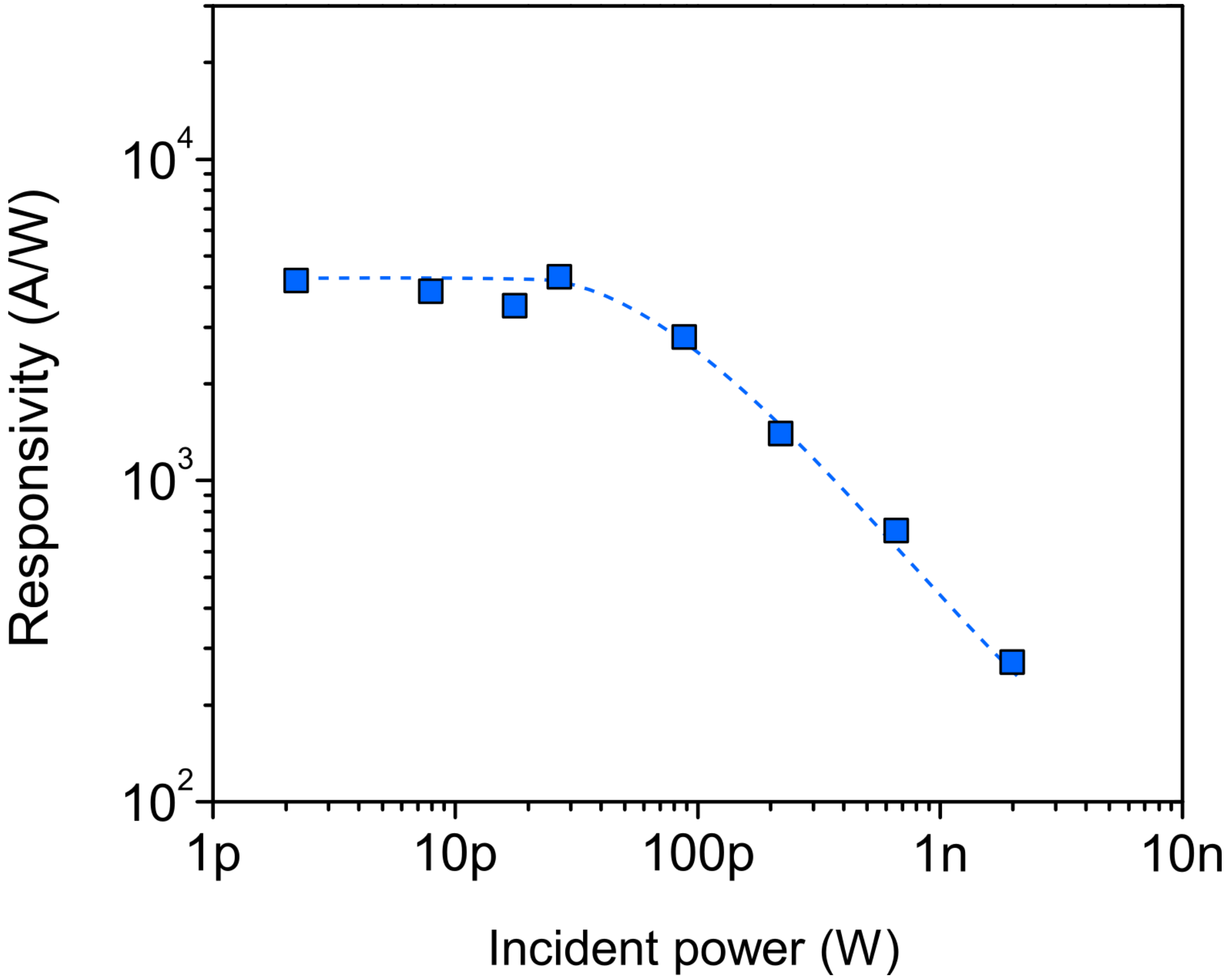

Supplementary Figure 15. Responsivity as a function of power. Responsivity as a function of incident light power. The measurement was taken under a 5 V applied bias, using a λ = 450 nm light source. As characteristic for photoconductive gain, the responsivity is inversely proportional to the light intensity and eventually approaches saturation at low optical powers. The dashed trace is a guideline for the eye.

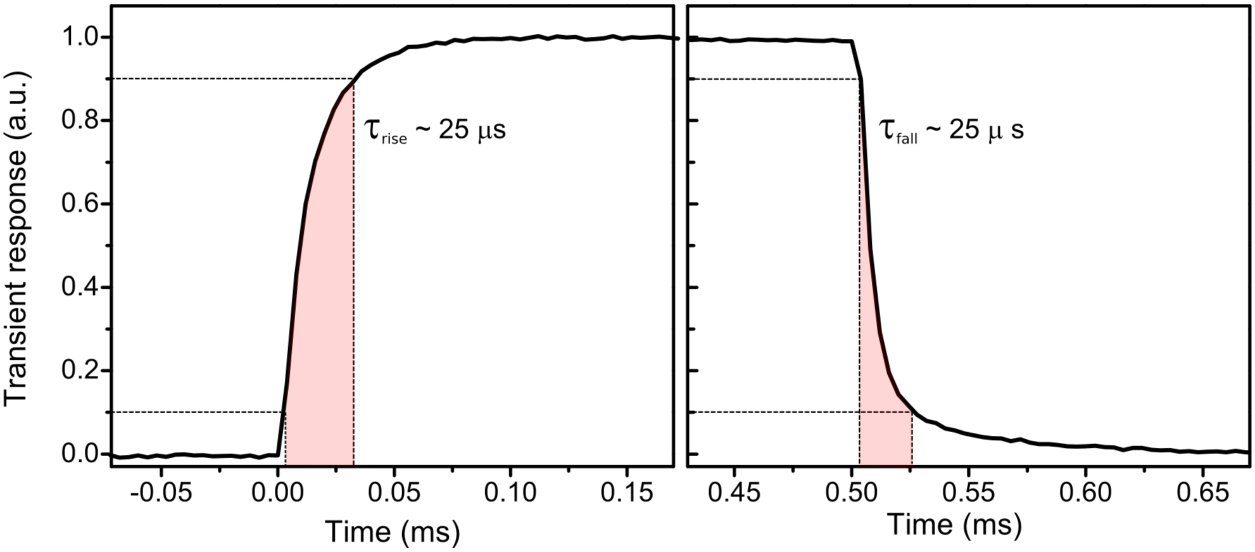

Supplementary Figure 16. Time response. Time response of the MSM photodetector. Rise and fall time are reported together for comparison; the characteristic time response does not change in the two cases. The values were obtained measuring the time the signal spends to rise (decay) from 10 (90) to 90% (10%) of the full wave amplitude. The measurement was taken using a 30 V applied bias and 3.8 μW light power (λ = 520 nm), pulsed at a frequency of 1000 Hz.

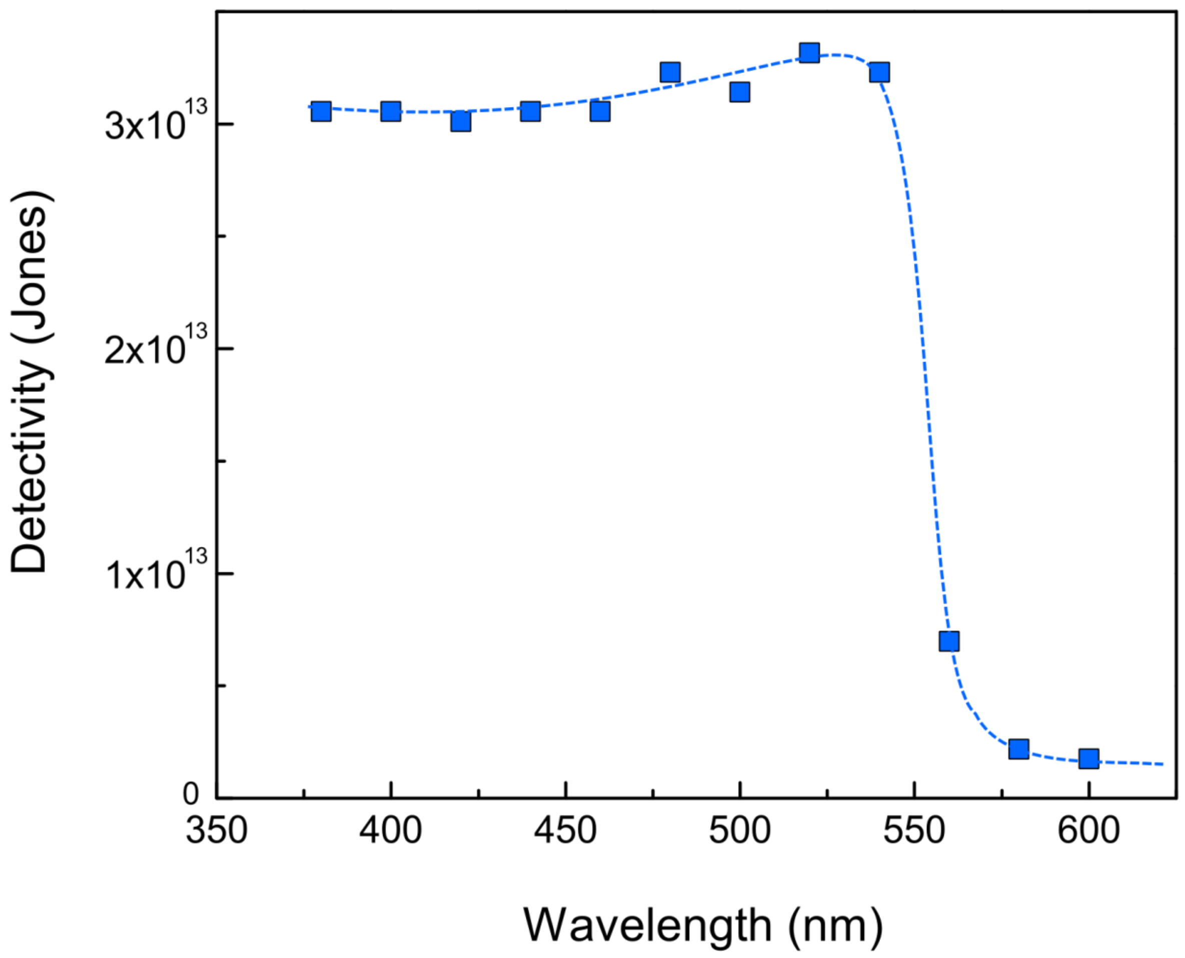

Supplementary Figure 17. Detectivity. Detectivity as a function of the light wavelength. The measurement was taken under a 5 V bias. The curve was evaluated using the dark current shot noise limit. The dashed line is obtained by using a b-spline interpolation of the data.

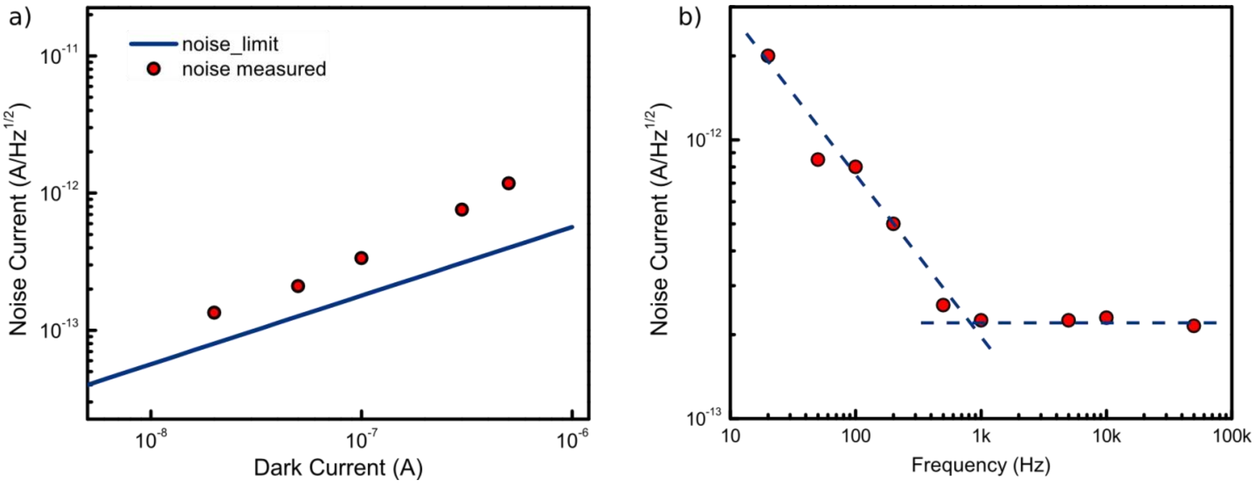

Supplementary Figure 18. Noise Current. a) Noise current as a function of the dark current. As expected the noise figure is dominated by the dark current shot noise. The noise current was measured using a Stanford Research SR830 lock-in amplifier at the frequency of 1 kHz. b) Noise current as a function of the modulation frequency. A pink noise component (~ *1/f* flicker noise) is found at low frequencies. The corner frequency is close to 1 KHz, well below the response time of the detector (~ 25 μs, Supplementary Fig. 15). The noise is measured for a dark current I_{dark} = 0.1 μA.

Supplementary References

1. Xing, G. *et al.* Long-range balanced electron- and hole-transport lengths in organic-inorganic CH3NH3PbI3. *Science* **342,** 344–7 (2013).

2. Dong, R. *et al.* High-Gain and Low-Driving-Voltage Photodetectors Based on Organolead Triiodide Perovskites. *Adv. Mater.* **27,** 1912–1918 (2015).

3. Dou, L. *et al.* Solution-processed hybrid perovskite photodetectors with high detectivity. *Nat. Commun.* **5,** 5404 (2014).

4. Fang, Y. & Huang, J. Resolving Weak Light of Sub-picowatt per Square Centimeter by Hybrid Perovskite Photodetectors Enabled by Noise Reduction. *Adv. Mater.* **27**, 2804-2810, (2015).

5. Guo, Y., Liu, C., Tanaka, H. & Nakamura, E. Air-Stable and Solution-Processable Perovskite Photodetectors for Solar-Blind UV and Visible Light. *J. Phys. Chem. Lett.* **6,** 535–539 (2015).

6. Lee, Y. *et al.* High-Performance Perovskite-Graphene Hybrid Photodetector. *Adv. Mater.* **27,** 41–6 (2014).

7. Hu, X. *et al.* High-Performance Flexible Broadband Photodetector Based on Organolead Halide Perovskite. *Adv. Funct. Mater.* **24, 24**, 7373–7380 (2014).

8. Li, D., Dong, G., Li, W. & Wang, L. High performance organic-inorganic perovskite-optocoupler based on low-voltage and fast response perovskite compound photodetector. *Sci. Rep.* **5,** 7902 (2015).
